# Dominance behaviour in a non-aggressive flatfish, Senegalese sole (*Solea senegalensis*) and brain mRNA abundance of selected transcripts

**DOI:** 10.1371/journal.pone.0184283

**Published:** 2017-09-06

**Authors:** Elvira Fatsini, Sonia Rey, Zohar Ibarra-Zatarain, Simon Mackenzie, Neil J. Duncan

**Affiliations:** 1 IRTA, Sant Carles de la Ràpita, Tarragona, Spain; 2 Institute of Aquaculture, Pathfoot Building, University of Stirling, Stirling, Scotland, United Kingdom; 3 CONACYT-UAN-CENIT, Calle 3 S/N, Ciudad industrial, Tepic, Mexico; Universidade de Vigo, SPAIN

## Abstract

Dominance is defined as the preferential access to limited resources. The present study aimed to characterise dominance in a non-aggressive flatfish species, the Senegalese sole (*Solea senegalensis*) by 1) identifying dominance categories and associated behaviours and 2) linking dominance categories (dominant and subordinate) with the abundance of selected mRNA transcripts in the brain. Early juveniles (*n* = 74, 37 pairs) were subjected to a dyadic dominance test, related to feeding, and once behavioural phenotypes had been described the abundance of ten selected mRNAs related to dominance and aggressiveness was measured in the brain. Late juveniles were subjected to two dyadic dominance tests (*n* = 34, 17 pairs), related to feeding and territoriality and one group test (*n* = 24, 4 groups of 6 fish). Sole feeding first were categorized as dominant and sole feeding second or not feeding as subordinate. Three social behaviours (i. “Resting the head” on another fish, ii. “Approaching” another fish, iii. “Swimming above another” fish) were associated with dominance of feeding. Two other variables (i. Total time occupying the preferred area during the last 2 hours of the 24 h test, ii. Organisms occupying the preferred area when the test ended) were representative of dominance in the place preference test. In all tests, dominant fish compared to subordinate fish displayed a significantly higher number of the behaviours “Rest the head” and “Approaches”. Moreover, dominant sole dominated the sand at the end of the test, and in the group test dominated the area close to the feed delivery point before feed was delivered. The mRNA abundance of the selected mRNAs related to neurogenesis (*nrd2*) and neuroplasticity (*c-fos*) in dominant sole compared to subordinate were significantly different. This is the first study to characterise dominance categories with associated behaviours and mRNA abundance in Senegalese sole and provides tools to study dominance related problems in feeding and reproduction in aquaculture.

## Introduction

Behavioural studies in fish have been used as a model background in the field of perceptive ethology/evolutionary psychology. Associating several cognitive traits (memory, kin recognition and learning, among others) with morphology, ecology and a variety of behavioural parameters could provide a general vision for cognitive ethology [1–4]. Moreover, the study of flatfish behaviour has attracted the attention of researchers due to their ecology, life history and adaptations. However, when considered in comparison with pelagic fish species, flatfish behaviour has received little attention. Similarly, studies have tended to focus on aggressive species and few social non-aggressive fish species have been studied. General aspects in the behavioural catalogue of flatfish have been described including feeding behaviour, locomotion, mimicry and spawning behaviour *reviewed in* Gibson [5]. However, dominance behaviour in flatfish has not been described. The understanding of flatfish dominance behavioural patterns would be very useful to comprehend the biology and ecology of flatfish species and for stock management in aquaculture and natural populations.

Senegalese sole (*Solea senegalensis*) is a typical flatfish species that undergoes a larval metamorphosis to form a flattened shape adapted for a benthic life. The species inhabits the sediments of the Mediterranean Sea and the Southern European Atlantic coast and in these areas forms an important fishery that is in decline. The combination of declining market supply and high market value has resulted in a rapid increase in Senegalese sole culture in Europe by more than 10 times from 2009 to a production of over 1,200 tons in 2015 [6]. However, at present the culture is unsustainable due to two of the main culture problems [7]. One is the variation in growth that results in large size dispersion during weaning and on-growing which complicates farming practices [8, 9]. The second issue is the complete failure in the spawning of cultured breeders (hatched and reared in captivity), which dictates that currently, the farming of Senegalese sole relies on captures of wild breeders that spawn in captivity. More specifically, cultured males exhibit a dysfunctional behaviour and consequently do not complete the reproductive courtship to fertilise eggs [10–12]. In addition to this problem with cultured males, wild Senegalese sole pairs (male and female) that reproduce in captivity show intra- and inter-annual fidelity and a few families from a few breeders (8–40% of the broodstock) dominate the offspring from a captive wild broodstock [13, 14]. In order to understand the reproductive behavioural dysfunction to control reproduction, the reproductive behaviour has been described (ethogram) [15]. This courtship was characterised as a complex set of behaviours that especially males executed to gain acceptance from a female to spawn in a pair [15]. In this context, cultured males did not engage with females and did not participate in reproductive behaviours [11, 12]. Furthermore, stress coping styles were identified for individual fish and compared between groups with different origins (cultured *vs*. wild) and reproductive success [16, 17]. However, stress coping styles were not related to origin or reproductive success [17, 18]. All these studies on growth, courtship and behavioural aspects found that Senegalese sole could be considered as a social non-aggressive species [8, 9, 12, 17]. Therefore, under aquaculture conditions Senegalese sole appear to exhibit dominance several aspects as in growth, feeding and reproduction. However, further studies are needed to understand inter-individual interactions in captivity of this species and particularly dominance as both of these problems, variation in growth and failed spawning, may be related to social interactions and dominance.

Dominance was defined as success in competitions over limited resources such as food, specific (or preferred) areas, shelter, mates, spawning areas and offspring [19]. Thus, in general, dominant animals could have better access to food and shelter, lower rates of predation and higher mating success [20] than subordinates. Conversely, subordinate animals might suffer chronic stress, immune depression, reduced disease resistance and lower reproductive success [21]. Aggressive behaviour was related to social hierarchies and competition in fish species such as zebrafish (*Danio rerio*), Nile tilapia (*Oreochromis niloticus*) or rainbow trout (*Oncorhynchus mykiss*) among other species [20, 22–24]. In fish, social hierarchies are often categorised by agonistic behaviours that are habitually registered through feeding contests or territoriality. Several variables could be measured to assess these social hierarchies and one of them is feeding behaviour [25] which has been associated with physiological indicators of stress [26, 27], aggression and mating success [28]. Currently, place preference test is also used for fish with several purposes, such as to observe active implication in reproduction and territorial dominance [29]. For example, Nile tilapia is a tropical fish species that build nests and defends a territory to attract females [30]. Thus, individuals choose between different cubicles depending on the stimulus motivating the visit, shelter or reproduction [29]. The knowledge of the natural preference and the competition for different compartments is essential to avoid a misleading interpretation of results [29, 31]. In addition, animals that acquire the dominant position in early life stages (juvenile) in fitness-related traits might expand this social status to reproductive success [32, 33]. Therefore, the use of feeding and place preference tests adapted to Senegalese sole could provide insights into how dominance is established and expressed in a social flatfish species and such information can provide tools to establish how social interactions lead to growth dispersion and / or suppression of reproductive success.

In addition, previous studies found that the differences in behavioural phenotype were associated to different levels of biological regulation measured in the transcriptome, showing the behaviour might be linked with differential gene transcription [34–36]. These studies are working towards an increased understanding of the behaviour—transcriptome linkage and the present study incorporated some of the identified transcripts, which will be briefly described here. Dominance behaviour has been associated with agonistic behaviour as aggression and some transcripts have been related to this conduct in zebrafish, for example: The serotonin receptor 1 A (5-Hydroxytryptamine receptor 1 A; *5-htr1a*), which inhibits serotonin (5-HT) firing, synthesis, release and turnover, has been associated with the control of several behavioural aspects including aggression [37, 38]. The tryptophan hydroxylase 1b (*tph1b*) that is involved in the synthesis of 5-HT [39] and solute carrier family 6 member 13 (*slc6a13*) that is a neurotransmitter transporter of *gamma*—Aminobutyric acid (GABA) were both related to aggression and impulsivity [40–42]. Lastly, the arginine vasopressin-induced protein 1 (*avplr1*), which is involved in the hypothalamo-neurohypophysial system (HNS) pathway and mineralocorticoid receptor (*mr*) implicated in hypothalamus-pituitary-interrenal (HPI) axis pathway, were also associated with aggressive behaviour in zebrafish [38]. Similarly, the following transcripts in zebrafish were associated with social status [43], *bdnf* and *c-fos*, which are markers of neuroplasticity [44] and *nrd2* that was associated with neurogenesis [45]. Finally, ETS translocation variant 5 (*etv5*) and nuclear receptor subfamily 4, group A, member 2 (*nr4a2*), which are implicated in the preservation and differentiation of dopaminergic neurons in tilapia (*Astatotilapia burtoni*) [46]. In addition, all of the mentioned transcripts have been related to social status in zebrafish [37, 38].

In this study, we provide insight into dominance and social interactions of captive Senegalese sole aiming to define dominance behaviour by relating behavioural patterns to feeding response and territory as well as defining mRNA abundance in association to the different dominance related behavioural responses.

## Material and methods

### Ethics statement

All experimental practises on fish that formed part of this study were in agreement with the Spanish and European regulations on animal welfare (Federation of Laboratory Animal Science Associations, FELASA) and approved by the Animal Ethics Committee of IRTA.

### Animal rearing conditions

Senegalese sole juveniles (~ 45 g) were provided by Stolt Sea Farm (Santiago de Compostela, Spain), different fish from the same batch were used for two successive years to conduct different behavioural tests. Fish were transported from A Coruña, where the facilities of Stolt Sea Farm are located, with a specialized transport for aquatic live animals. Fish were maintained at the Research Centre facilities of IRTA, in St. Carles de la Ràpita, North East Spain and distributed in two 10 m^3^ fiberglass tanks with natural photoperiod (using artificial lighting) and seasonally simulated external temperatures (40°62’82.42”, 0°66’09.37”/ 9–32°C) maintained within the range of 9–20°C with a recirculation system (IRTAMar^®^) that recirculated + 400% and renewed 10% of the water daily. Sole were fed *ad libitum* five days per week with balanced feed (LE-3mm ELITE, Skretting, Co.). A total of six identical 400 L fiberglass tank were used, two tanks were used for acclimation (17–25 fish or 2.5–4.8 kg per tank) and four tanks for the experiments. One week before dyadic tests started, animals were moved from a large holding tank and acclimated to the 400 L fiberglass tanks. All fish were tagged with a passive integrated transponder (PIT) tag (ID—100A, Unique Trovan-Zeuss; Madrid, Spain) and photo identified individually. Individual markings in the photographs were matched with the corresponding PIT Tag for later identification in the video images. The acclimation and experimental tanks were in a recirculation system to control the temperature and water quality in order to reduce environmental variation. During the tests the temperature was maintained at 15°C with a recirculation system (IRTAMar^®^ described above). The animals were fasted for 48 hours prior to the experiment.

### Behavioural studies

#### Video recording analyses

Digital cameras (Square black and white CCD camera, model F60B/N80-50G, KT&C Co. Ltd., Korea Technology and Communications Korea, supplied in waterproof housing by Praesentis S.L. Barcelona, Spain) were used to film the dyadic and group dominance tests. One camera per experimental tank was placed above the tank to give a view of the entire tank. Four cameras (one per experimental tank) were connected to a digital video recorder (model XMOTION-304H by Praesentis, S.L). The videos were analysed in two ways, a continuous analysis of 2 hours of the behaviours between the two fish in the dyadic tests and a discrete analysis of behaviours on particular frames in the group tests. The different behaviours (*defined in the dominance test section*) were registered and their frequency was annotated for further statistical analysis.

#### Preliminary dyadic test

A preliminary dyadic test was performed with twelve early juvenile sole (when fish weight was approximately 100 g) according to Huntingford *et al*., [47] to decide which test and setup was the most appropriate to characterise dominance behaviour in Senegalese sole. Animals were provided with different experimental cues that were visual (animals were separated by a transparent screen through which the animals could see each other), chemical (animals were kept apart by an opaque screen with holes through which the sole could exchange chemical cues) and isolated (sole were separated by an opaque screen without the possibility to see and smell each other). The different tests were performed at different daylight hours (morning or night). When preliminary results (S1 File) were analysed Senegalese sole did not show differences in behaviour due to the different experimental setups, and all fish resumed feeding and ate normally. Therefore, visual and chemical cues between separated fish did not appear to affect feeding. Considering this, the isolation approach was selected to provide a basal condition, where animals had equal status before testing for dominance over a limited resource, which is an approach that has been previously used in other fish species [25, 48].

#### Dominance tests

**Fig 1:**
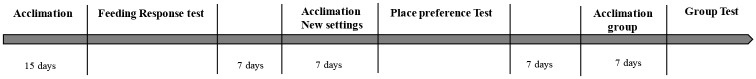
Experimental chronogram of the different test conducted in Senegalese sole juveniles. Experimental chronogram of the different dominance behavioural tests conducted on late Senegalese sole juveniles.

Three different behavioural tests (two in pairs (dyadic) and one in group) were performed for Senegalese sole juveniles to test for dominance. Only one of the behavioural tests, feeding response in a dyadic test, was applied in early juveniles (*n* = 74; 100.4 ± 10.6 g). All three tests were applied to late juveniles (*n* = 34; 287.0 ± 30.4 g) and the resting time between tests was 7 days to allow for full recovery, return to basal conditions and to avoid learning and conditioning processes plus 7 days of acclimation to the new setting conditions (*see* Fig 1 for set-up and time line of the experiments). In all dyadic tests the fish were size-matched and paired (< 10% in weight between animals).

#### Dyadic tests

1) Feeding response after a dyadic test. Two size-matched sole taken from different acclimation tanks, to avoid previous competition, were kept apart overnight (19:00 to 8:00 = 13 hours) by a dark polyvinyl chloride (PVC) wall. The wall was removed the following morning at 08:00 and their behaviours were continuously recorded for the first two hours. After two hours, fish were hand-fed with approximately 1% of the total biomass of the experimental tank (two sole) and the individual that ate first was classified as dominant. The individual that ate afterwards or did not eat was classified as subordinate. Behaviours were individually registered and their frequency of occurrence was recorded by video monitoring. The behaviours recorded were: “Approaches”, “Swimming above another” (SAA), “Rest the head” (RTH), “Displacement”, “Burying” and finally order of feeding “Feeding” that was used to define dominance (categorical variable) (Table 1: ethogram of behaviours).
2) Competitive behaviour for a preferred area: place preference test. For the place preference test the settings of the tank were modified. First a bottom was created in the tank by placing twenty white tiling pieces (measuring 24 x 11.5 x 1.5 cm each and the same colour as the bottom of the tank) and second, one tile was removed to provide a space that was filled with sand. The area with sand was the standard size of one fish and so just one fish could comfortably occupy this area. Sole individuals used for this test had an extra week of acclimation to those novel objects: the sand and the tiling. The purpose of this test was to create a preferred area with sand (in nature, sole live buried in sand), which just one fish could occupy and, therefore, dominate that area (*see* S1 Fig. for set-up details). Previous studies have shown that sole prefer sandy areas [49]. The experimental tank was set up with PVC opaque/grey divisions that separated the two fish from each other and from a third area that contained the sand. The same pairs of sole that were previously used in the feeding test were also used in the place preference test and this enabled the place preference behaviour of the sole to be analysed in relation to the dominant and subordinate status observed in the feeding test. Fish were physically isolated (in separate acclimation tanks) and were introduced into the experimental tank at 19:00 and left overnight. The dividers were removed the following morning at 08:00 (after 13 hours of isolation) allowing the fish to see each other and access the restricted preferred area (sand). The behaviour of the fish in relation to the preferred sandy area was recorded continuously for 24 hours for further video analysis of recorded behaviours (Table 1). Red night lighting was used that allowed recording and observation of the sole behaviour. Red light was from fluorescent lights covered with a red filter. The intensity of the red light was adjusted to approximately 5 lux at the water surface. Previous studies demonstrated that this red light did not affect behaviour or physiological parameters during the night recordings [50]. The variables registered in this test were measured in minutes and regarding to the preferred area (sand). The variables were “Total time” in the preferred area (TT), “Initial time” to occupy the sand (Ti), “Final time” to occupy the sand (Tf) and order: “first” or “last” (categorical variable) (Table 1).

**Table 1:** Ethogram of different behaviours registered in Senegalese sole.

Behaviours, parameters and Index	Acronym	Test	Description
Approaches		1	A fish approaches another fish without making physical contact.
Swimming above another	SAA	1	A fish swims near and above another fish.
Rest the head	RTH	1	A fish rests the head on another fish. This behaviour is performed resting the head on different parts of the body.
Displacement		1	A fish displaces another fish making contact, for example, swimming directly towards the another fish to make direct contact.
Burying		1	A fish makes a wave type movement of the body and lateral fins starting from the head to the tail that in substrate would bury the animal. This behaviour has been associated with fear or escape, burying to rest and to reject other fish.
Feeding		1	A fish eats the pellets provided registered as “Yes” or “No”.
Initial time or latency	Ti	2	The total time that each fish remains in the preferred area (sand) during the first 2 hours (minutes) of the experiment.
Total time	TT	2	The total time of each animal remains in the sand during the 24 hours test (minutes)
Final time	Tf	2	The total time that each fish remains in the preferred area (sand) during the last 2 hours (minutes) of the test.
Order position		2	Order that the fish were observed in the preferred sand area at the beginning and end of the experiment, “First” was the animal which entered the sand first. “last” was the animal in the sand when the test finished after 24 hours. The same fish could have both positions.
Feeding order		3	Order which fish ate in the group test. Fish that ate first was 1, second fish to eat was 2 etc.
Rest the head index	RTH Index	3	The number of times that a fish rests the head on another fish minus the number of times other fish rested the head on the fish under consideration.
Swimming above another index	SAA Index	3	The number of times that a fish swims closely above another fish minus the number of times other fish swam above the fish under consideration.
Position “before feeding” index	POSITB Index	3	Index per fish per day = ((*position 1* x *“y”*) + (*position 2* x *“y”*) + (*position 3* x *“y”*) + (*position 4* x *“y”*) + (*position 5* x *“y”*) + (*position 6* x *“y”*))/6; “*y*” = frequency of each position during the hour before feeding. Position was registered every 5 minutes (12 frames).
Position “after feeding” index	POSITA Index	3	Index per fish per day = ((*position 1* x *“y”*) + (*position 2* x *“y”*) + (*position 3* x *“y”*) + (*position 4* x *“y”*) + (*position 5* x *“y”*) + (*position 6* x *“y”*))/6; “*y*” = frequency of each position during the hour after feeding. Position was registered every 5 minutes (12 frames).
Feeding index		3	The mean of the feeding order registered each of the 4 days for each individual.

Ethogram of the different behaviours, parameters and index registered (each different behaviour performed were counted and registered) for the three dominance tests performed (feeding response, place preference test and group test).

#### Group test

3) Dominance in groups. Immediately after the place preference test were finished, four groups of six fish (24 fish in total) were randomly selected and placed in four 400 L experimental tanks. As with previous tests the fish were given a one-week recovery period followed by a one-week acclimation period that allowed the group to acclimate and to establish inter-individual hierarchies (Fig 1). After this period of acclimation, each group of fish was recorded to analyse the social interactions in the group. The different behaviours (Table 1) were recorded for 2 hours (every 5 minutes analysing a total of 24 frames) one hour before fish were fed and one hour after. The social behaviours recorded in the group test were “Rest the head” (RTH) and “Swimming above another” (SAA). The behaviours were registered in counts and different indexes were calculated (actions among animals). The test was performed in four consecutive days and the same behaviours for each of the groups of fish were recorded to test for consistency and repeatability among days. Fish was visually individually identified. An automatic feeder was placed in a corner of each tank and feed was delivered directly to the bottom of the tank through an 18 mm PVC tube to provide a single feed delivery point (*see* S2 Fig for set up details). Fish were fed with approximately 1% of the total biomass of the experimental tank. This single feed delivery point set-up is known to trigger territoriality and feeding competition among the individuals and dominant fish, at higher positions in the social hierarchical rank, tend to monopolise the feeding point [51–54]. The exact physical position of the fish in relation to the feeding point was recorded every 5 minutes before (1 hour = 12 frames) and every 5 minutes after (1 hour = 12 frames) feeding events (24 frames in total). The positions of the fish were ranked in order of the fish from the feeding point, i.e. the fish closest to the feeding point was ranked 1, second closest was ranked 2 etc. The ranked positions from the analysed frames were used to calculate the indices that were termed: “Position before feeding index” (POSITB index) and “Position after feeding index” (POSITA index) per fish and per day (Table 1). Mean of every index was calculated per fish over the 4 days. In addition, the “Feeding order” (‘pecking order’) for each day of the experimental period was registered to check for consistency over time.

### RNA isolation, complementary DNA synthesis and quantitative real-time polymerase chain reaction assay

Thirty of the seventy-four early juveniles were randomly chosen, fifteen fish from each category (dominant/subordinate) and were sacrificed with an overdose of MS-222 (tricaine methanesulfonate; Acros-Organic, New Jersey, USA). Whole brains were extracted, frozen in dry ice and stored at -80°C for molecular analysis. The RNA was extracted using TRI Reagent RNA Isolation Reagent following manufacturer’s instructions (SigmaAldrich). The cDNA was synthesised using 1 μg of total RNA and oligo dT (20) in 20 μl reactions and the SuperScript^®^ III First-Strand Synthesis SuperMix 50 rxn kit following the manufacturer’s protocol (Invitrogen, Life technologies, USA). Primers were designed using Primer 3 [55] in 3UTR region. Before performing the qPCR, primers were validated by conventional PCR using a cDNA pool from several samples randomly chosen to analyse the primers. MyTaq^™^ HS Mix (Bioline) was used to run the conventional PCR with the following conditions: initial activation step at 98°C for 1 min, followed by 35 cycles: denaturation at 95°C for 10s, annealing at Tm (58–60°C) gradient conditions for 15 s and extension at 72°C for 15 s. Primer efficiency was evaluated by serial dilutions to ensure that it was close to 100% performing *real time* PCR. Target transcripts with implication in neuroplasticity, neurogenesis and brain activation (*bdnf*, *c-fos* and *nrd2*) [43, 44, 56], related to differentiation of dopamine neurons (*nr4a2* and *etv5)* [46, 57], behavioural responses and aggression (*5-htr1a*, *tph1b*, *avplr1* and *slc6a13*, *mr*) [37–39, 42] were analysed by quantitative PCR (*q*PCR) (*see* primer design in Table 2). The *q*PCR was run using a Biometra Optical Thermocycler (Analytik Jena, Goettingen, Germany) in 96-well plates in duplicate 20 μl reaction volumes containing 10 μl of Luminaris Color HiGreen *q*PCR Master Mix (Thermo Scientific), 1 μl of the primer corresponding to the analysed gene (10 pmol), 3 μl of RNA/DNA water free and 5 μl of cDNA in its corresponding dilution. Furthermore, amplifications were carried out with a systematic negative control (NTC; no template control) containing no cDNA. Standard amplification conditions contained an UDG pre-treatment at 50°C for 2 min, an initial activation step at 95°C for 10 min, followed by 35 cycles: 15 s at 95°C, 30 s at the annealing Tm and 30 s at 72°C. Results were normalised using three housekeeping genes ubiquitin (*ubi52*), glyceraldehyde-3-phosphate dehydrogenase (*gapdh2*) and elongase factor 1 alpha (*eef1a*) and applying a geometric average [58]. The mRNA abundance for each gene was determined using the Pfaffl method [59].

**Table 2:** Primers used in this study as possible dominance biomarkers for early Senegalese sole juveniles.

Gene	Gene name	Amplicon size	Accession Number	Primer (5' 3')
**C-FOS**	*c-fos*	175	unigene4094	F-CTGGAGTTCATTCTGGCTGC
				R-TTGAGGTGAATGTTGGCTGC
**Brain-derived neurotrophic factor**	*bdnf*	154	unigene54354	F-ACTCGTTTGAAACATCCGGC
				R-CAGACAGGGTGAGTGGAGAA
**Neurogenic differentiation factor 2**	*nrd2*	396	unigene1444	F-TTATCAGTGTGCGCGTCTGT
				R-TTCAGTTCGTCGTACACGGG
**ETS translocation variant 5**	*etv5*	165	unigene42532	F-CACTCTGATGCCAACGTTCA
				R-CAGCGACAAGAACACGGAG
**Nuclear Receptor Subfamily 4, Group A, Member 2**	*nr4a2*	187	unigene55326	F-TCTCCCGAGTTTCAGCACTT
				R-CCCAGAGTGAGCCATCATTT
**5-hydroxytryptamine receptor 1A**	*5-htr1a*	180	unigene35339	F-GCTGGCTGCCCTTTTTCATC
				R-CCGCATGTGGTTATTGCCTG
**Arginine Vasopressin-induced protein 1**	*avplr1*	153	unigene17371	F-TGTTGTCGACCACTCACTCA
				R-TGAAAGGTTGTGCGTGTCTG
**Tryptophan hydroxylase 1b**	*tph1b*	218	unigene62116	F-GGAAGCTGCGAGCATATGGA
				R-GAAGGGACGCTTGATGTTCT
**Solute carrier family 6 member 13**	*slc6a13*	166	unigene3332	F-GTTAACTGCCTGTCCCGTCA
				R-ACCGTGTAGTGTGAACGAGG
**Mineralocorticoid receptor**	*mr*	204	unigene4626	F-GCACTCCACATGCACTCAAA
				R-CCTTTGCCCTGTAGTCTTGC

Gene, gene name, size accession number (SoleaDBv4.1) and primer sequence are indicated.

### Statistical analysis

#### Behaviour

All means are presented as mean ± standard error of the mean (SEM). In the case of paired tests (early and late juveniles), the coefficient of variation (CV % = SD/mean*100) that represented the inter-individual sole variability were calculated for each category (dominant and subordinate) and compared. All data was tested for normality with the Shapiro-Wilks test and data that failed was corrected with a Log_10_ transformation. To reduce variables and define behaviours that best represented dominance, a Principal Component Analysis (PCA) with adequacy of Kaiser-Meyer-Olkin test and Bartlett’s test of spherity with Varimax rotation was applied. In the case of early juveniles, a Spearman’s correlation analysis was run for the feeding response test between the variable “Feeding” (if the animals ate or not) and those variables, which were representative in the PCA run for this test. Student’s t-test was performed to compare means of counts of different behaviours of dominant and subordinate fish.

For the group test Kendall’s concordance coefficient (0.43 fair concordances) was calculated for each fish/behaviour index (RTH, SAA, POSITA and POSITB) to check the concordance among the 4 days for the same fish in each group. A “*k*” means cluster was applied to variables chosen by Kendall’s concordance coefficient for all groups. As there was concordance within the individuals in their behaviours over the 4 days for the index RTH, SAA and POSITB the mean of each index was calculated. Thus, Student’s t-test was applied to check the differences between dominants and subordinates. Statistical analyses were performed using SPSS Statistics 19.0 software (IBM Co., Hong Kong) and GraphPad Prism 6 software (GraphPad Software, Inc.) and *P* < 0.05 was used to establish significant differences.

#### q- rtPCR

The outliers of the different categories (dominant/subordinate) of the corrected ratio of every mRNA were extracted using the Tukey’s test formula (*k* = 1.5). The data was transformed to Log_10_ (*var* + 1) and Student’s t-test was applied to compare mRNA abundance between individuals grouped as dominant or subordinate. The threshold was considered at 0.3 related to pooled control animals simulating the population, where values under that threshold indicated down-regulation and over, up-regulation. The pooled control animals were from the same batch of the experimental sole used for this study and were acclimated to the same tanks as the experimental fish. Nevertheless, these fish were fed normally and were not used for any experimental process to obtain objective data imitating the usual conditions of the facilities. Raw data from both dominance behaviour and mRNA abundance are available in *figshare* (DOI: 10.6084/m9.figshare.4964990).

## Results

### Senegalese sole behavioural responses in dominance tests

Senegalese sole were classified as dominant or subordinate according to their feeding response (in dyadic and group feeding tests), the first fish to feed were classified as dominant and fish that fed second or did not feed at all were classified as subordinate. In general, subordinate individuals showed more variability in responses than dominant fish, which indicates that subordinate fish exhibited more variation between individuals.

Comparing the behaviours, “Approaches”, “Swimming above another” (SAA) and “Rest the head” (RTH), the dominant early juveniles (*n* = 36 of 74) displayed less variability within these behaviours (Approaches = 9.6 ± 1.4 counts; CV = 86.5%; SAA = 5.8 ± 1.2 counts; CV = 119.1%; RTH = 4.5 ± 0.6 counts; CV = 76.5%) than subordinates (*n* = 38 of 74) (Approaches = 5.5 ± 1.0 counts; CV = 109.2%; SAA = 2.5 ± 0.6 counts; CV = 141.1%; RTH = 2.6 ± 0.5 counts; CV = 105.4%). Whilst, the two behaviours, “Burying” and “Displacement”, presented a similar variability for both dominant and subordinate fish (Burying = 4.4 ± 0.5 counts; CV = 65.8%; Displacement = 1.0 ± 0.2 counts; CV = 150.3% for dominants and Burying = 4.6 ± 0.5 counts; CV = 66.0%; Displacement = 0.5 ± 0.2 counts; CV = 187.6% for subordinates respectively).

The trend in late juveniles was similar to that observed in early juveniles, with similar levels of variation in the behaviours and dominant late juveniles (*n* = 17 of 34) also displayed less variability (Approaches = 18.9 ± 2.6 counts; CV = 56.4%; SAA = 27.3 ± 9.5 counts; CV = 144.3%; RTH = 9.1 ± 1.4 counts; CV = 65.9%) than subordinates (*n* = 17 of 34) (Approaches = 12.1 ± 2.5 counts; CV = 83.8%; SAA = 12.3 ± 3.6 counts; CV = 120.3%; RTH = 4.1 ± 0.9 counts; CV = 91.5%).

The same pairs from the dyadic feeding tests were used in the place preference test and feeding response defined dominance was used in the place preference test to also define which fish were dominant and subordinate. In the place preference, dominant late juveniles spent more time in the preferred sand area at the end of the test and had less variability (Initial time (Ti) = 25.5 ± 9.2 min; CV = 149.6%; Total time (TT) = 377.1 ± 71.1 min; CV = 77.7%; Final time (Tf) = 60.7 ± 11.4 min; CV = 77.8%) than subordinates (Ti = 33.6 ± 11.7 min; CV = 144.2%; TT = 302.9 ± 93.9 min; CV = 127.8%; Tf = 38.4 ± 11.1 min; CV = 119.6%).

#### Observed feeding behaviour (MAPs in Senegalese sole)

The feeding behaviour of Senegalese sole was similar to that observed for other flatfish species with a defined “predation cycle or modal action patterns (MAP)”: searching, encountering, capturing and ingesting the food [5, 60]. In the present study, Senegalese sole both early and late juveniles, showed MAPs associated with feeding behaviour at the moment food was introduced into the experimental tank (*see* S2 File). Therefore, sole classified as dominant interacted first with the food and blocked other interactions. Furthermore, individuals that ate and were considered as dominant sole, interacted with the food in the first 10 minutes of food delivery starting with a “searching” MAP that consisted in slow creeping movements over the bottom, moving the head from side to side in short actions while slowly approaching the food. At the moment an individual detected the food the fish orientated and swam rapidly straight to it and started to protect the food and the area in which the food was delivered (*direct observation*).

### Dominance parameters selection

#### Individual tests

**Fig 2:**
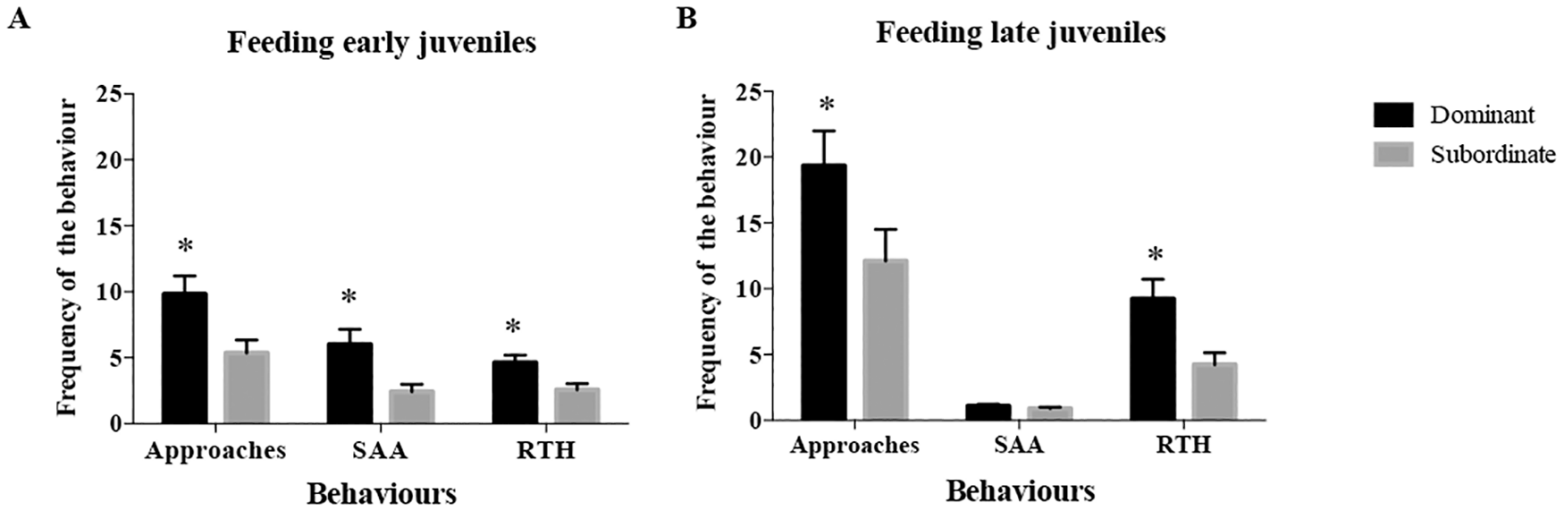
Frequency of the behaviours analysed in early and late juveniles of Senegalese sole. Number of movements were counted during the 2-hour test that dominant and subordinate (A) early juveniles and (B) late juveniles Senegalese sole exhibited the three behaviours “Approaches”, “Swimming above another” (SAA) and “Rest the head” (RTH). Data was shown in Mean ± SEM (*n* = 74 early juveniles; *n* = 34 late juveniles). An * indicates a significant difference (*P* < 0.05).

In early juveniles, three behaviours, “Approaches”, “Swimming above another” (SAA) and “Rest the head” (RTH) (Table 1) were grouped together (PCA, KMO (0.667), Bartlett’s test (*P* < 0.001) and *X*^2^ (133.523); S3 Fig). These three behaviours expressed more than 51% of total variance after the principal component analysis. Spearman’s correlations demonstrated that those selected behaviours were weakly correlated to feeding response (Approaches: r_s_ = 0.372, *P* < 0.001; RTH: r_s_ = 0.358, *P* = 0.005; SAA: r_s_ = 0.4, *P* < 0.001; S4 Fig) In relation to feeding order, animals were classified as dominant or subordinate as described above. There was a pair that did not feed, and in this case both fish were considered subordinates. Consequently, the counts of the three behaviours were significantly different between dominants and subordinates (Approaches: *t* = 2.675, *df* = 72, *P* = 0.01; RTH: *t* = 2.814, *df* = 72, *P* = 0.01; SAA: *t* = 2.877, *df* = 72, *P* = 0.01; Fig 2A). Dominant sole displayed significantly more approaches, resting the head and swimming above another than subordinate sole.

The association between behaviours and dominance was similar for the late juveniles. The PCA of all the variables from the feeding response and place preference test that were applied to late juveniles extracted three components that explained 72% of the variance of the data (KMO (0.6), Bartlett’s test (*P* < 0.001) and *X*^*2*^ (116.806); S5 Fig). The three behaviours, Approaches, RTH and SAA were grouped together as the first principal component (PC1). These three behaviours were also grouped together for the early juvenile sole establishing that the feeding response test applied to two different groups of sole of different ages and size gave similar consistent results. The second (PC2) and third components (PC3) for the late juveniles were both related to behaviours from the place preference test. The PC2 was formed by the variables; total time each fish occupied the preferred sand area during the last two hours of the test (Final time—Tf) and which fish was in the sand when the test finished at 24 hours (last position—last). PC3 consisted of; the time each fish first occupied the sand during the first two hours (Initial time—Ti), total time each fish was in the sand during the entire 24-hour test (Total time—TT) and which fish was first to enter the sand area (first position—first) (Table 1).

The counts of behaviours “Approaches” (*t* = 2.036, *df* = 31.69, *P* = 0.05) and RTH (*t* = 2.894, *df* = 26.30, *P* = 0.008) were significantly higher in dominants than subordinates in late juveniles. However, SAA (*t* = 1.083, *df* = 30.94) was not different between dominants and subordinates (Fig 2B).

**Fig 3:**
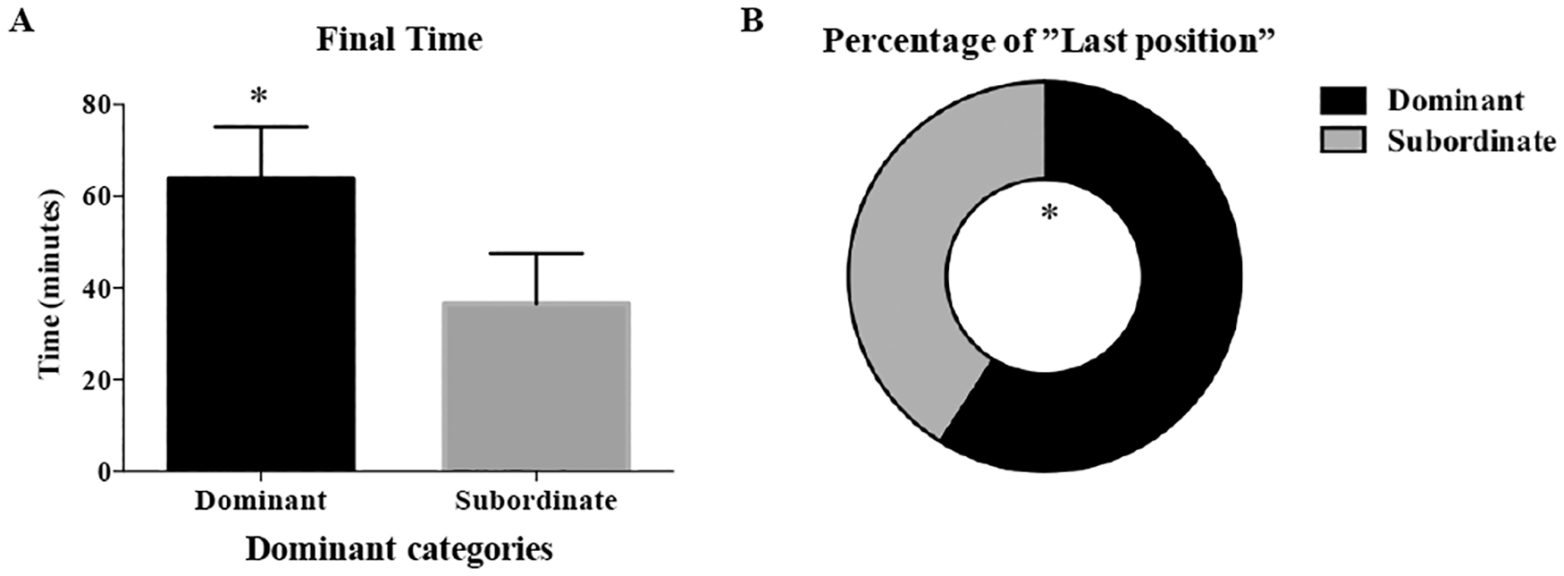
Place preference test in late juvenile sole (*n* = 74). (A) Time in minutes during the last two hours of the test that dominant and subordinate Senegalese sole were in the preferred sand area (Tf). (B) The proportion of dominant and subordinate fish that were in the preferred sand area when the test finished (last). An * indicates a significant difference (*P* < 0.05).

The variables that formed the second component were significantly different between dominant and subordinate fish classified by feeding response test, thus sole classified as dominant (in the feeding test) spent more time in the preferred sandy area during the last two hours of the test (Tf: *t* = 2.186, *df* = 16, *P* = 0.044; Fig 3A) and dominated the area when the test finished after 24 hours (*X*^*2*^ = 5.674, *P* = 0.017; Fig 3B). In summary, the dominant fish that ate first spent more time in the preferred area over the last two hours of the test and monopolised the sand, showing that the final position was indicative of a dominant status. All results were analysed from size-matched pairs and, therefore, variability due to unequal size distribution was avoided. Thus dominance parameters were observed in each pair representing pairs of similar weight and length.

#### Group test

**Fig 4:**
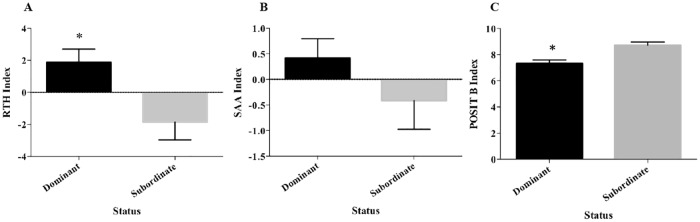
Mean behavioural indices for dominant (*n* = 12) and subordinate (*n* = 12) late juveniles sole in the group-test according to Kendall’s concordance coefficient. (A) “Rest the head Index” (RTH Index); (B) “Swimming above another Index” (SAA Index) and (C) “Position before feeding” (POSITB Index). An * indicates a significant difference (*P* < 0.05).

In the group analysis, “Rest the head index” (RTH Index), “Swimming above another index” (SAA Index), “Position of the fish in relation to the feed delivery tube before feed” was delivered (POSITB) and “Feeding Order” (from 1^st^ to 6^th^ position) (Table 1) showed agreement (0.43) according to Kendall’s concordance coefficient (W) among the 4 days for each group (S1 Table). The animals in the different groups were analysed as a single population and the Kendall’s concordance coefficient demonstrated that the inter-groups index were consistent. In addition, “*k*” means cluster classified animals in two clusters that represented dominant and subordinate animals. The two clusters grouped the same animals according to feeding RTH, SAA and POSITB Index indicating concordance of these behaviours in the different groups. Therefore, 12 fish were classified as dominant and 12 as subordinates. Student’s t-test applied in those index to compare the significant differences between dominants and subordinates for different behaviours showed that RTH Index (*t* = 2.659, *df* = 10.46, *P* = 0.015; Fig 4A) and POSITB Index (*t* = 3.779, *df* = 21.57, *P* = 0.001; Fig 4C) were significantly different between dominant and subordinate groups. However, the SAA Index (*t* = 1.231, *df* = 19.35; Fig 4B) was not different between dominant and subordinate fish. To summarize, the fish considered dominant more often occupied positions closer to the feed delivery tube even before the food was provided (place preference test was covered with those positions) and rested the head (RTH) in any part of the body more often than the subordinate fish. The group analysis was performed with the four groups as a population consisting of individuals of different sizes (simulating the cohabitation in nature). However, no relationship was found between fish size and the repetition of the different dominance parameters observed. For example, in one of the groups, the weight range was from 195.7 to 310.6 g and the two smallest animals presented more actions in RTH and POSITB and presented more consistency in their data than the largest individual throughout the experimental period.

### Brain mRNA abundance in relation to dominance

**Fig 5:**
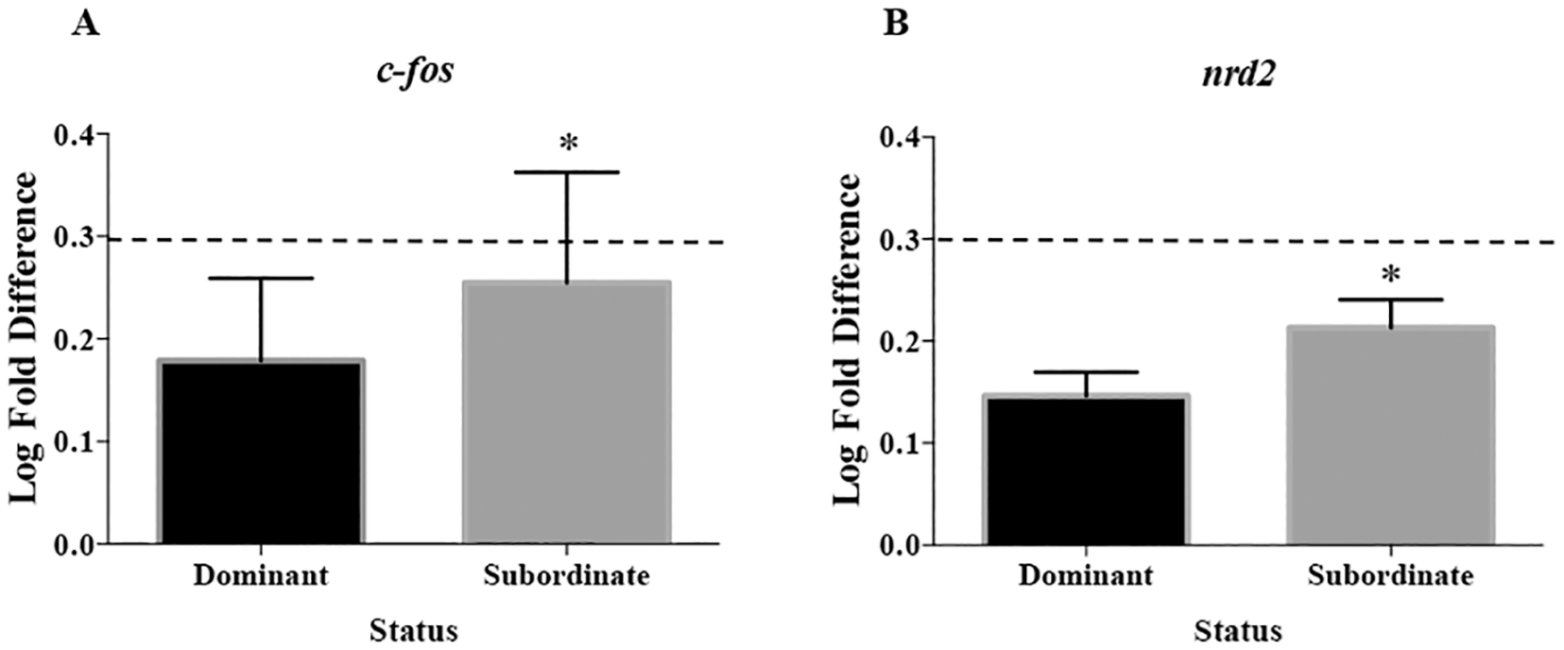
mRNA abundance of dominant (*n* = 15) and subordinate (*n* = 15) early juvenile sole. Expression for two genes (A) *c-fos* and (B) *nrd2* shown as mean ± SEM in Log (*var* + 1) transformation. Dashed line at 0.3 indicates the value of a pooled control simulating the population; values under 0.3 indicate down-regulation. An * denotes a significant difference (*P* < 0.05).

Only two of the ten (Table 2) mRNAs tested exhibited significant differences in mRNA abundance between dominant and subordinate sole (Table 3). Both transcripts, *c-fos* (*t* = 2.014, *df* = 20.13, *P* = 0.041; Fig 5A) and *nrd2* (*t* = 1.861, *df* = 27.15, *P* = 0.047; Fig 5B), were down regulated in both dominant and subordinate fish in relation to pooled controls that represent the entire range for the population. This down-regulation was more pronounced in dominant sole than in subordinate individuals. The *nr4a2* mRNA presented marginally different abundance (*t* = 1.987, *df* = 22.56).

**Table 3:** mRNA abundance in Log (*var* + 1) fold difference for the several mRNAs analysed in dominant (*n* = 15) and subordinate (*n* = 15) early juvenile Senegalese sole.

	Log (*var* + 1) Fold difference		
Genes	Dominant	Subordinate	p-value
*bdnf*	0.3506 ± 0.02955	0.3394 ± 0.03255	0.8005
*c-fos*	0.1787 ± 0.02148 a	0.2548 ± 0.03255 b	0.0410*
*etv5*	0.3305 ± 0.03719	0.3440 ± 0.03775	0.7950
*nr4a2*	0.2052 ± 0.01116	0.2481 ± 0.01850	0.0592
*mr*	0.2614 ± 0.02190	0.2884 ± 0.03130	0.4860
*5-htr1a*	0.1753 ± 0.01933	0.1825 ± 0.01596	0.7760
*tph1b*	0.3759 ± 0.07202	0.3270 ± 0.07368	0.6388
*avplr1*	0.2884 ± 0.02222	0.2981 ± 0.03550	0.8180
*nrd2*	0.1465 ± 0.02297 a	0.2132 ± 0.02745 b	0.0471*
*slc6a13*	0.2070 ± 0.01213	0.2607 ± 0.02284	0.1261

Data was shown mean ± SEM.

An * and different letters indicate a significant difference (*P* < 0.05) in mRNA abundance between dominant and subordinate sole. Means less than the pooled control population value of 0.3 were considered down-regulated.

## Discussion

The present study has shown that Senegalese sole can be categorized for different social status (dominant and subordinate): (a) when competition was observed between pairs and in groups, (b) in relation to both feeding response and place preference tests, and (c) at different juvenile stages, from early juvenile, when fish weight was approximately 100 g, to late juvenile that weighted approximately 300 g (considered as pre-adult). Across the three tests (dyadic feeding, dyadic place preference and group) the same behaviours were associated with social status. Dominant fish compared to subordinate fish displayed a significantly higher number of the behaviours “Rest the head” (RTH) on another fish, “Approach” another fish and “Swim above another” (SAA) fish. This is the first description of behaviours associated to social status in a non-aggressive fish and/or a flatfish species. Other studies on social status in fish have been conducted on round fish that display aggressive behaviours [24–26, 29, 31]. For example, previous studies in rainbow trout demonstrated that after a period of isolation the fish that ate first won the subsequent contest, showed more aggressive behaviour and were subsequently categorized as dominant individuals [25]. This agrees with the present study where both animals were isolated before the dyadic behavioural tests and the sole that ate first exhibited a higher frequency of the behaviours RTH, Approaches and SAA and also dominated preferred spatial zones (sand or feed delivery point).

As mentioned in the introduction two of the main problems for sole culture are a large variation in growth [8, 9] and a complete failure in the spawning of cultured breeders [11, 12]. Therefore, the identification of different social status and associated behaviours in sole have considerable implications in studies directed to tackling effects of dominance in culture and ecological studies for sole and flatfish.

### Feeding and growth

A major problem during grow out reported in Senegalese sole is a large variation in growth resulting in a wide dispersion of sizes and the requirement of frequent grading [8, 9]. In the present study, dominant sole clearly had advantages in access to feed and both ate first and dominated the feed source (i.e. space where feed was delivered). Therefore, one theory would be that dominance could explain the size variation observed in cultured stocks and that this study might be fundamental for future research into the dominance of feeding and effects on growth in captive conditions. In agreement with this, aggression in feeding behaviour is the most common method used to establish hierarchies and in different species the dominant fish have often been the larger individuals [61]. However, juvenile Senegalese sole did not show any aggressive behaviour at different stocking densities when fed according to the biomass [8] or in the dominance tests (present study). In the present work, larger body size was not identified as a factor associated to dominant fish in the present study and consequently dominance behavioural parameters specifically RTH, SAA and Approaches were not associated to animal size. These observations would suggest that dominance is probably a contributing factors, but that dominance alone may not be responsible for the growth variation and other factors, such as life strategy, appetite and feeding behaviour, should be considered.

Life strategies with differences in appetite can be compared to stress coping styles that range from a proactive life style where animals take risks to forage for food, suffer higher predation, grow faster and reach puberty at a younger age [17, 62]. In comparison the opposite extreme a reactive life style where animals take less risks, exhibit less foraging for food, higher survival, slower growth and reach puberty at an older age [17, 63]. Proactive and reactive coping styles have been described in all stages of Senegalese sole, larvae [64], juveniles and adults [17] and proactive sole were shown to grow faster and reach puberty earlier than reactive sole [18]. Furthermore, previous studies have shown that benthic larvae of Senegalese sole have more activity than juveniles, and that juveniles present more activity than adult sole under certain conditions [17, 18]. Therefore, different life strategies among individuals may modify changes in activity and feeding to contribute to the size variation observed in cultured stocks.

The feeding behaviour observed during the present study was similar to that described in the common sole (*Solea solea*) and other species such as turbot where visual and olfactory cues are important in searching for food [65–67]. Performance of the different parts of the predation cycle (search, encounter, capture and ingestion) can vary between the species. In common sole feeding behaviour and boldness (associated in some fish species with aggression and social status such as in cichlid fish [68]) were related to feed intake using isolation and group tests [69] explaining variability in growth where proactive fish appear to have better feeding tactics in captivity. Feeding order in fish could be similar to the pecking order found in other farmed species (poultry, pigs, cows, etc.), pecking order is established at a younger age, and remains stable along their life determining the social status and hierarchies. In addition, pecking order has been associated with different activities such as feeding, drinking, and mating [70, 71]. This could be the explanation for the consistent feeding behaviour in groups among the four days, considering that the individual behaviour can affect the variability of the group in captivity.

### Reproduction

As explain in the introduction cultured males (born and reared in captivity) presented a dysfunctional behaviour and consequently did not complete the reproductive courtship to fertilise eggs [10–12]. Wild breeders held in captivity reproduce normally in captive condition, however, in a broodstock a few reproductive pairs show intra- and inter-annual fidelity and dominate the reproductive output [13, 14]. A description of the reproductive behaviour in captive wild breeders has shown that the behaviours Rest the head (RTH) and “Approaches” were essential courtship behaviours that males had to execute towards females to achieve reproductive success [15]. Immediately before spawning, a male was observed to approach the female and rest the head on the female. Once the male was accepted, the female swam from the bottom to initiate the coupled swim to the surface to spawn. Particularly, RTH appeared to be a behaviour that indicated a pair, the female accepting the male and the male protecting or dominating the female from other males. Therefore, the behaviours RTH and “Approaches” indicate dominance in feeding and place preference (present study) and acceptance / dominance of a partner in mate selection and reproduction [15]. Dominance across different resources including mate selection has been described in aggressive round species such as Nile tilapia [30], Ambon damselfish (*Pomacentrus amboinensis*) [72, 73], Mozambique tilapia (*Oreochromis mossambicus*) [74] and social mammals such as meerkats [32] or European rabbits [33], where dominance status started in early life stages and were persistent until adult phase. The present study provides the bases for further research to determine if the behaviours RTH and “Approaches” indicate dominance across feed, territory and mate selection.

### Place preference test

Place preference test consisted of two areas as choice possibilities, where the animal associates one of those areas as preferred for a particular purpose (hide, shelter, mating, among others) [75, 76]. In our study, there was a clear preference for the area with substrate by all experimental sole. However, the objective of this test was to observe the behaviour of paired Senegalese sole in response to a preferred area (sand) simulating a limited resource. Preference by dominant sole regarding territory was identified taking the different times of every individual dominating the substrate. Therefore, dominant sole spent more time at the end of the test (last two hours of test) and were more often present in the sand at the end of the 24-hour test (last position). The final two hours of the place preference test coincided with the initiation of a daytime resting period, when the dominate sole would occupy the preferred space by burying into the sand to rest. This kind of diurnal activity with daytime burying into sand and resting was first described in common sole [77]. Therefore, subordinate fish spent less time at the end of the test and entered the sand earlier in the test or did not enter at all. This was in agreement with other fish species with different biology and ecology such as Mozambique tilapia where substrate is important in different contexts [78]. Another example of the use of substrate is the Nile tilapia, where animals were isolated and individuals could choose different compartments where the gravel-enriched compartment was the most visited [29].

The movement performed by sole when in contact with the sand was burying. The burying behaviour has three objectives, burying in to rest, hiding and to displace another sole (when the burying is displayed under or on top of another sole) [15]. Kruuk (77) described the same behaviour for common sole having several functions, such as burying to help mimic the sediment (camouflage enables flatfish to avoid predators and remain hidden to prey), to initiate a resting period and to avoid currents. However, this behaviour was not extracted as a representative parameter in feeding response test in early juveniles, it was a key behaviour to determine the time parameters of place preference test in late juveniles. Previous studies demonstrated that habitat preference increased the level of territorial protection or dominance displayed by brown trout (*Salmo trutta*) [79]. In contests dominant animals defended and displayed more aggression in order to dominate their preferred territory. In our study, when both sole coincided in the sand the sole in the upper position normally the dominant individual rested the head (whatever part of the body) on the sole in the bottom position. This behaviour would not be considered as an aggression as the other animal was not injured, but it could be a harassment tactic. So, the RTH parameter extracted in the feeding response test was also associated with RTH behaviour in the place preference test. However, the situation of both animals coinciding in the sandy area was not usually observed in fact only in two of the seventeen pairs studied. In the case of the group test the place preference was analysed according to the position regarding the feeder supplier area. Intriguingly, the POSITB Index corroborated that animals that dominated the area to which feed was delivered were the animals that ate in the first positions, being stable during the experimental period. This situation is commonly observed in cultivated fish due to domestication, which depending on the fish species is performed in a different manner achieving different tactics according to the food delivery [80].

### Brain mRNA abundance in association to dominance behaviour

Several sets of genes related to different behavioural processes were analysed in the present study. However, just two mRNA transcripts related to neurogenesis (*nrd2*) and neuroplasticity (*c-fos*) were differently expressed between dominant and subordinate sole. Besides, *nr4a2* one gene which encode NR4A2 a receptor associated with differentiation of dopamine neurons [46, 57] was marginally different. These three transcripts presented down-regulation in both categories (dominant and subordinate) compared to control group (*see* Fig 5 for *nrd2* and *c-fos*) and dominant fish exhibited greater down-regulation than subordinate fish. In some cases, the presence of the same low profile in both dominance categories, dominant and subordinate, results in the adaptive response to slight social stress in comparison to the undisturbed animals [81]. This could be the explanation of why in our study animals of both dominance categories showed down-regulation, therefore, dominant and subordinate sole would present the same pattern of social stress being more pronounced in dominant sole.

In several animal models for social studies such as rodents, social subordination has been associated to reduced neurogenesis [82]. The mRNA transcript related to neurogenesis was *nrd2* that is essential for the survival of specific populations of neurons and neuronal differentiation in mice [83, 84]. This transcript has also been related to neural plasticity in zebrafish where the expression was higher in winners than losers in dorsal telencephalic area [45]. Neuroplasticity has also been associated with social interactions in fish where the profile of immediate early genes (IEG), including *c-fos*, in zebrafish exhibited acute changes in the pattern of expression due to different social status [45, 85]. Another example was found in tilapia (*Astatotilapia burtoni*); males of this species can change social status between subordinate and dominant in relation to the presence or absence of a larger dominant male in the same territory. This behavioural change between subordinate and dominant was shown to be accompanied at the molecular level with changes in the transcript IEG *c-fos* [86]. In our study, the pattern of transcript abundance was distinct where dominant and subordinate sole presented lower *c-fos* abundance than a representative population pool corroborating that neuroplasticity was possibly associated with social behaviour in this species.

In the present study, *5-htr1a* jointly with other aggression-related genes such as *tph1b*, *avplr1* and *slc6a13*, *mr* presented similar mRNA abundance between dominant and subordinate sole contrarily to other fish species where dominance was determined by aggressive behaviour [38, 87]. However, as previously mentioned Senegalese sole is considered a non-aggressive species and these transcripts, related to social status and aggression in other species, may not have a role in social status in the non-aggressive Senegalese sole.

## Conclusions

In conclusion, this is the first study using dyadic contests in a flatfish species to describe and determine social status (dominant/subordinate) when resources were limited. This study reports upon individual differences in dominance behaviour in Senegalese sole for the first time. This fish is considered as a non-aggressive species and accordingly different non-aggressive dominance behavioural parameters (Rest the head, Approaches, and Swim above another) were described in relation to dominance of feeding and territory. Those dominance behaviours were consistent in the different juvenile stages (early and late juveniles) and additionally, those parameters were reliable when applied to sole in groups. Both dominant and subordinate juvenile Senegalese sole exhibited specific and significantly different levels of abundance of two brain mRNAs that used to be associated to neurogenesis and neuroplasticity. Nevertheless, future work would be necessary to completely assess the relationship between brain gene transcription and differences in dominance profiles in this species. These results are highly relevant for the fish farming industry of Senegalese sole in order to tackle problems that appear to be linked to dominance in the culture system and form the bases for work to improve culture conditions by understanding the behavioural profile of these animals. This essential understanding of hierarchical distribution in the population will be linked to methods to ensure future reproductive success.

## Supporting information

S1 Fig Experimental tank set up used for the place preference test (with sand) in pairs. **A** Preferred area (sand) and **B** white tiles forming a false bottom characterized the novel conditions. **C** Water inlet. **D** Water outlet.
(TIF)

S2 Fig Group experimental tank set up used for feeding response and point feed delivery. **A** PVC tube to deliver the food. **B** Water Inlet. **C** Water Outlet. Different position areas (1–6) were shown by point lines.
(TIF)

S3 Fig Principal component analysis of the different behaviours registered during the “feeding response test” in pairs. The three variables “Approaches, SAA and RTH” were grouped together and explained the 53% of the variance of the data. KMO (0.667), Bartlett’s test (*P* < 0.05) and *X*^2^ (133.523).
(TIF)

S4 Fig Spearman’s correlations from the three variables “Approaches, SAA and RTH” with feeding. (**) correlation was significant *P* < 0.05.
(TIF)

S5 Fig Principal component analysis of the different behaviours registered during the feeding dominance test and place preference test (sand) in pairs. The three variables “Approaches, SAA and RTH” and the “TF and last” explained the 56% of the variance of the data in two different components. KMO (0.6), Bartlett’s test (*P* < 0.05) and *X*^2^ (116.806) (SPSS 19.0 IBM Statistics).
(TIF)

S1 Table Classification of the different variables in groups according to Kendall’s concordance coefficient (W) for every group. (*P < 0.05) level of significance.
(DOCX)

S1 File Raw data of preliminary results in dyadic pairs of early Senegalese sole juveniles. (XLSX)

S2 File Modal action patterns related to feeding in late Senegalese sole juveniles. (WMV)
