# Supplementary figures and images for: Dominance behaviour in a non-aggressive flatfish, Senegalese sole (Solea senegalensis) and brain mRNA abundance of selected transcripts
Source: PLoS One. 2017 Sep 6;12(9):e0184283. doi: 10.1371/journal.pone.0184283 (PMC5587333; doi:10.1371/journal.pone.0184283)

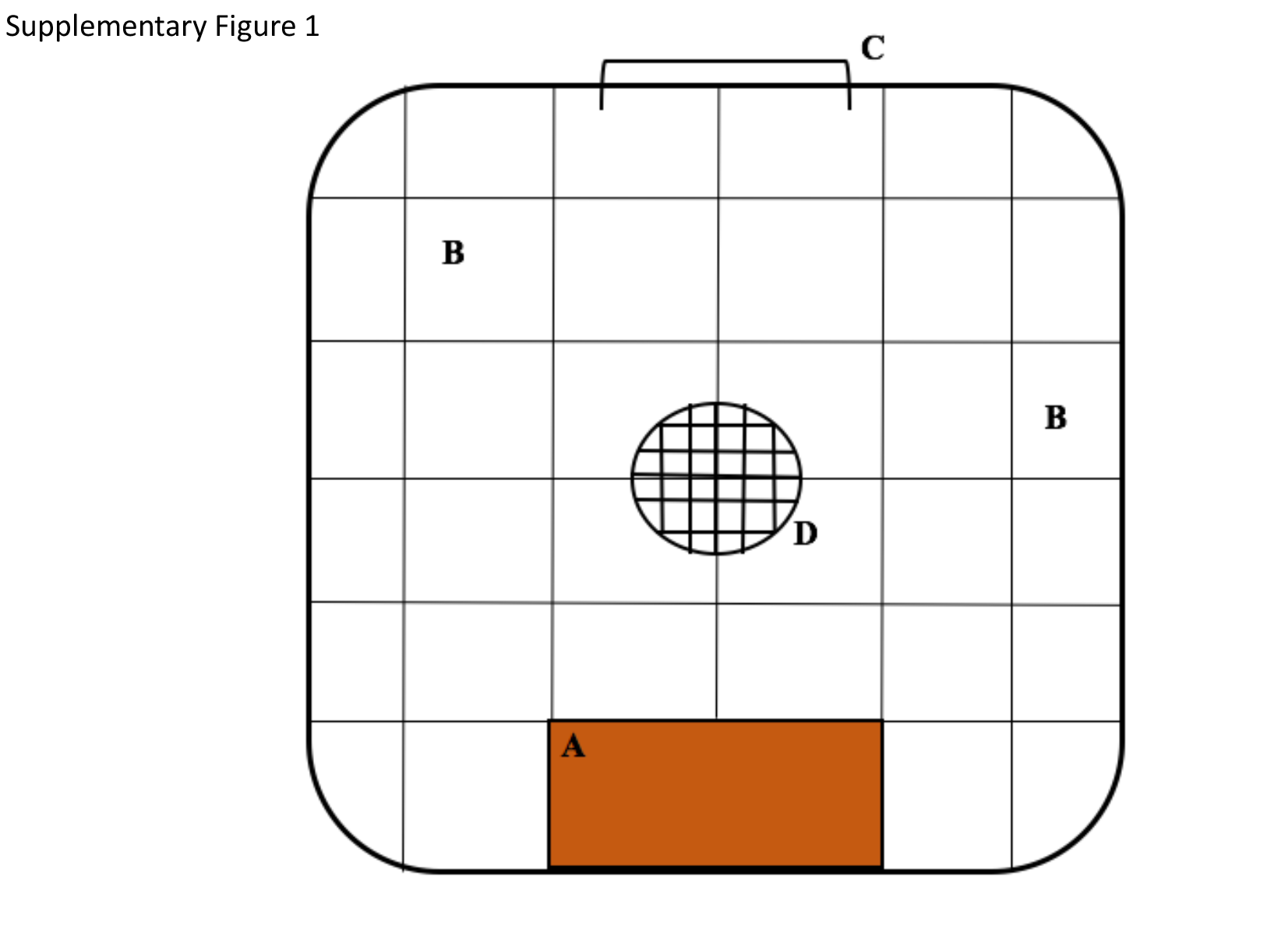

Supplement: S1 Fig — A Preferred area (sand) and B white tiles forming a false bottom characterized the novel conditions. C Water inlet. D Water outlet. (TIF) [file pone.0184283.s001.tif]

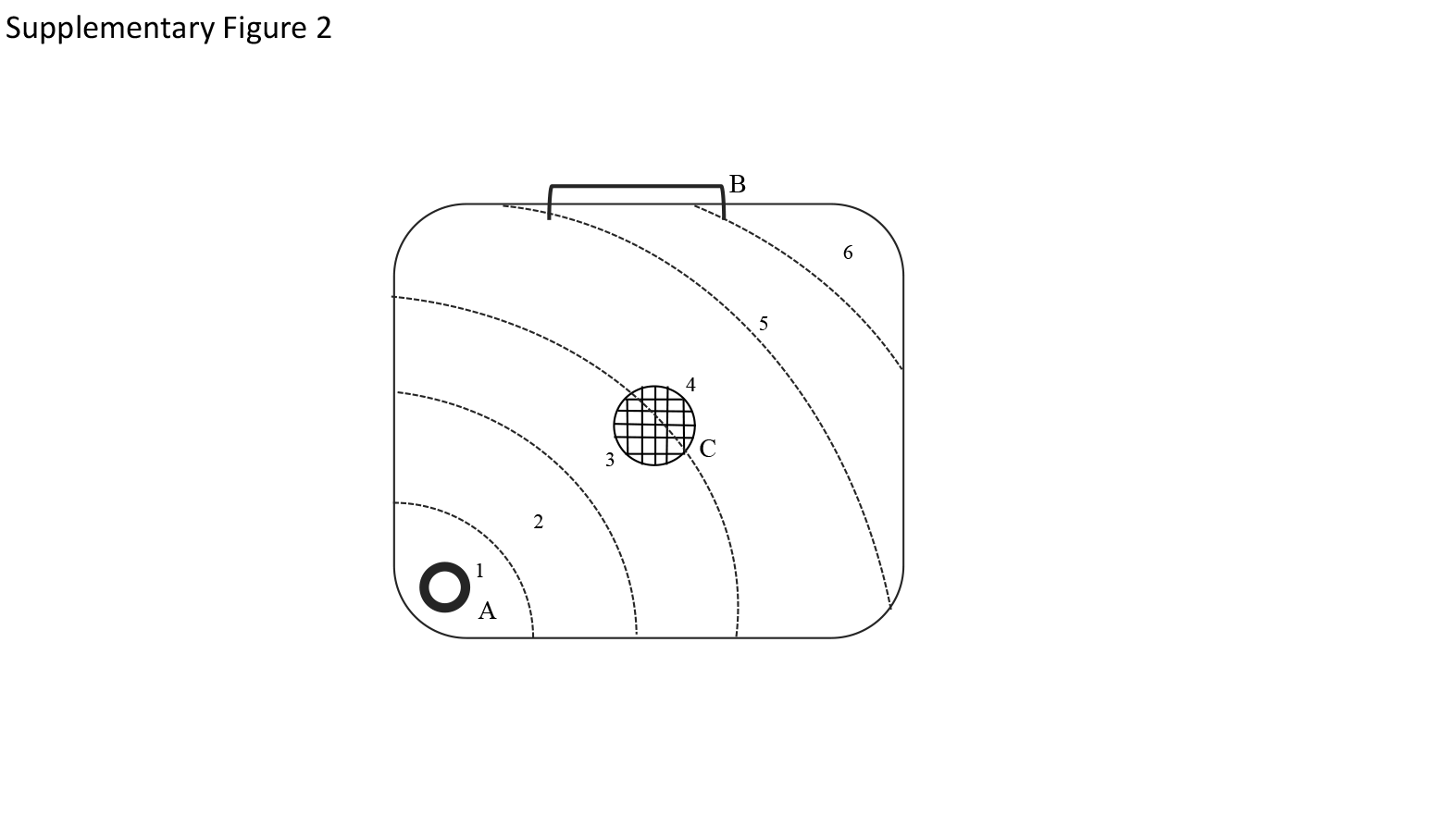

Supplement: S2 Fig — A PVC tube to deliver the food. B Water Inlet. C Water Outlet. Different position areas (1–6) were shown by point lines. (TIF) [file pone.0184283.s002.tif]

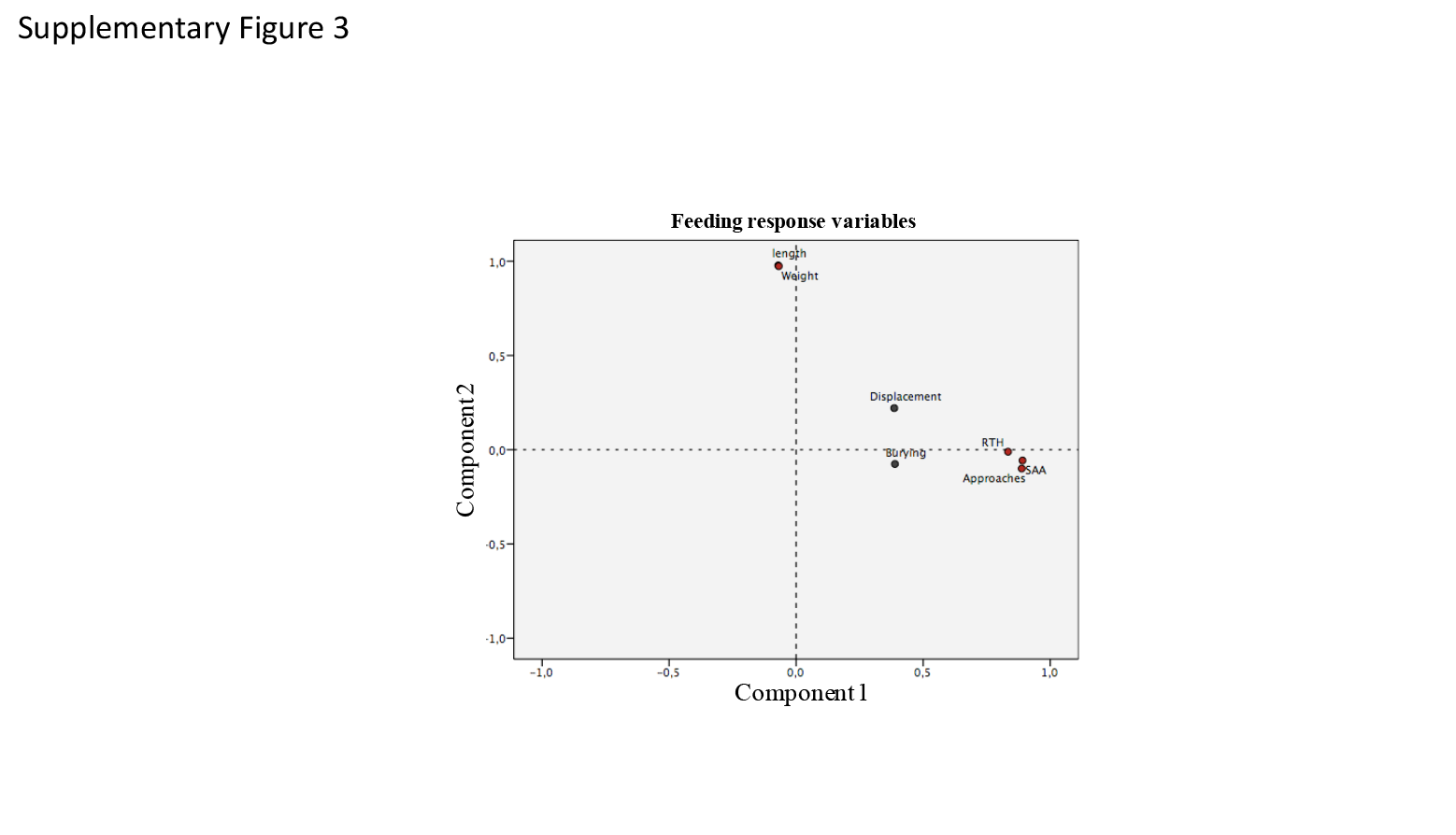

Supplement: S3 Fig — The three variables “Approaches, SAA and RTH” were grouped together and explained the 53% of the variance of the data. KMO (0.667), Bartlett’s test (P < 0.05) and X2 (133.523). (TIF) [file pone.0184283.s003.tif]

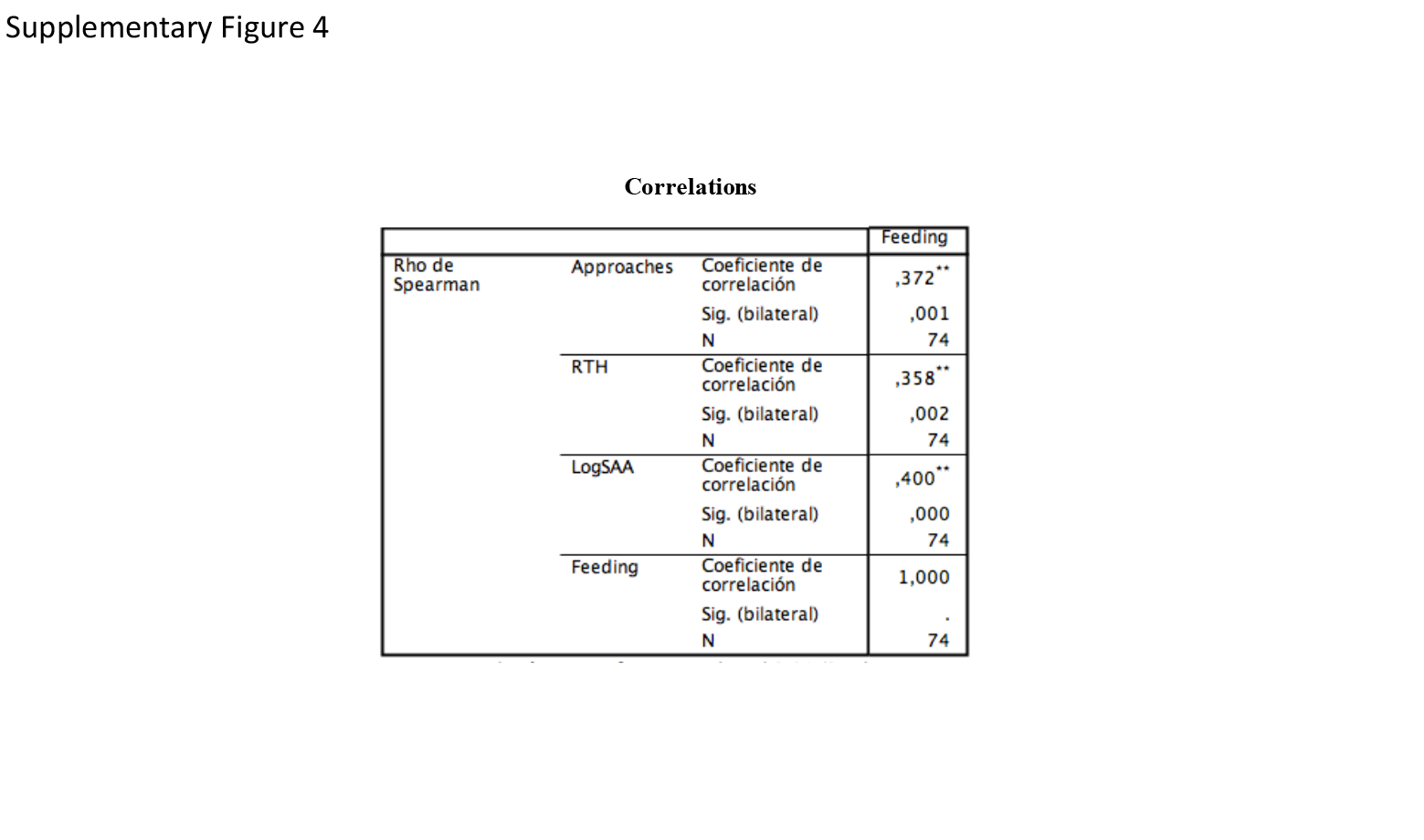

Supplement: S4 Fig — (**) correlation was significant P < 0.05. (TIF) [file pone.0184283.s004.tif]

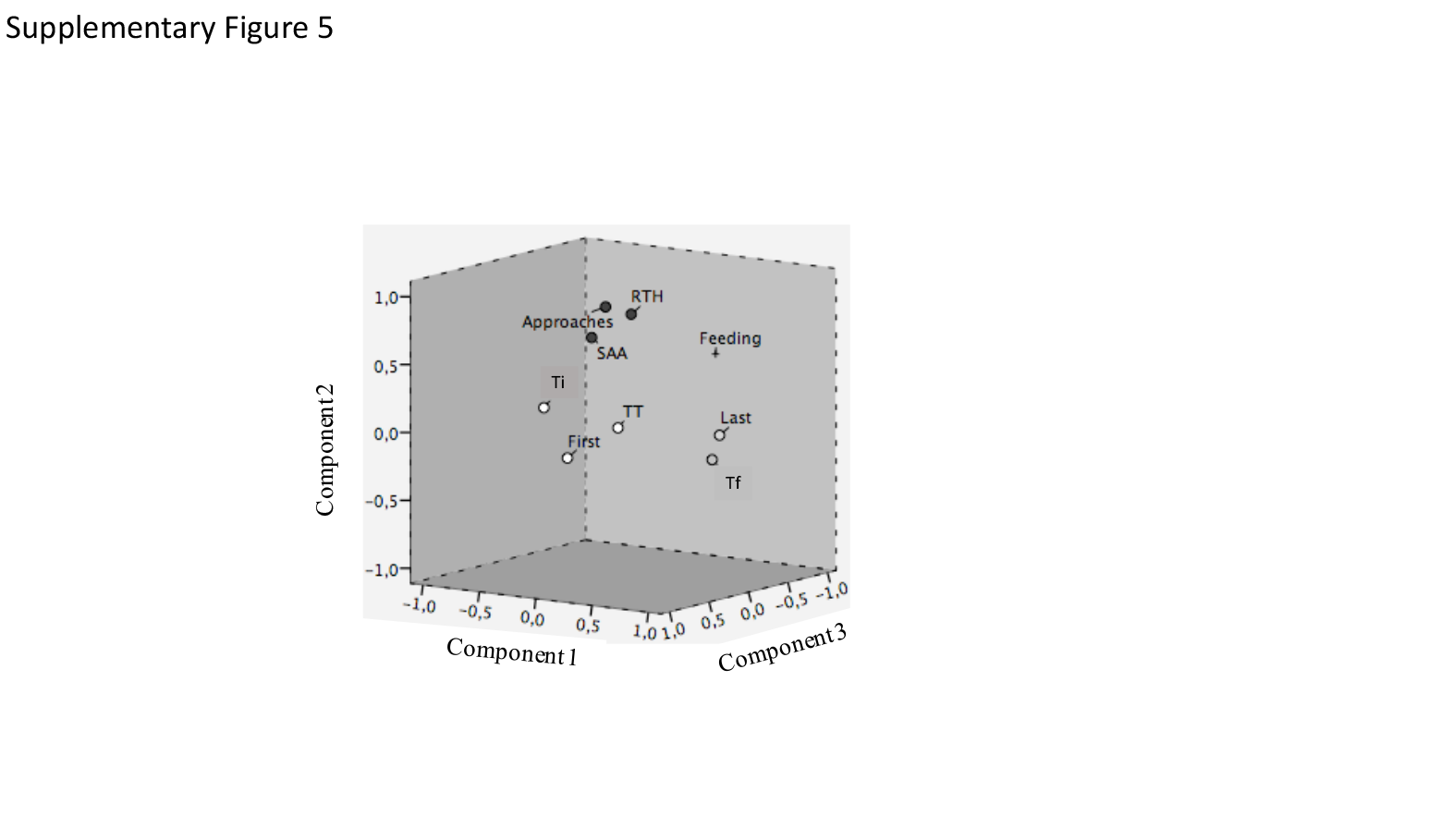

Supplement: S5 Fig — The three variables “Approaches, SAA and RTH” and the “TF and last” explained the 56% of the variance of the data in two different components. KMO (0.6), Bartlett’s test (P < 0.05) and X2 (116.806) (SPSS 19.0 IBM Statistics). (TIF) [file pone.0184283.s005.tif]
