# Supplementary material for: Dominance behaviour in a non-aggressive flatfish, Senegalese sole (Solea senegalensis) and brain mRNA abundance of selected transcripts
Source: PLoS One. 2017 Sep 6;12(9):e0184283. doi: 10.1371/journal.pone.0184283 (PMC5587333; doi:10.1371/journal.pone.0184283)
Supplement: S1 Table — (*P < 0.05) level of significance. (DOCX) [file pone.0184283.s006.docx]

S1 Table. Classification of the different variables in groups according to Kendall’s concordance coefficient (W) for every group. ((*) level of significance).
